# Supplementary material for: Recurrence of Equinus Foot in Cerebral Palsy following Its Correction—A Meta-Analysis
Source: Children (Basel). 2022 Mar 2;9(3):339. doi: 10.3390/children9030339 (PMC8947726; doi:10.3390/children9030339)
Supplement: Supplementary file 1 [file children-09-00339-s001.zip › children-1586153-supplementary.pdf]

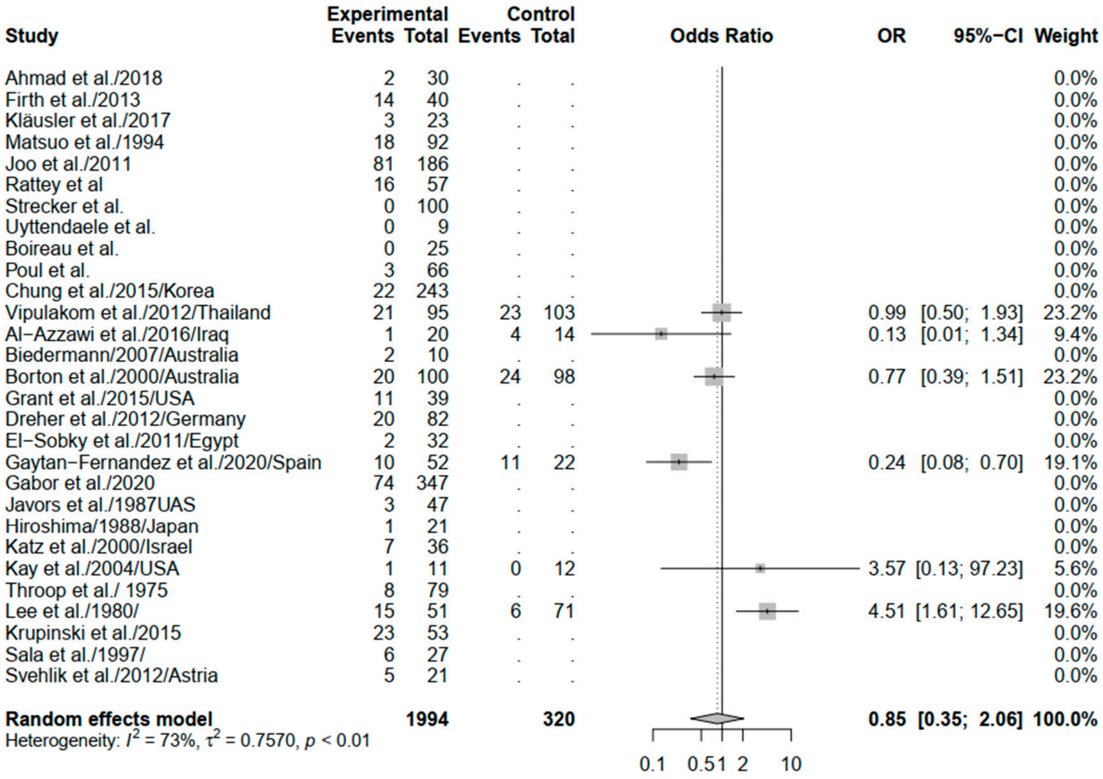

Figure S1. Forest plot of the risk of equinus recurrence among studies with two comparison groups.
